# Quality of Life After Surgical Treatment of Head and Neck Paragangliomas

**DOI:** 10.1002/hed.70084

**Published:** 2025-11-06

**Authors:** Christina Sauter, Philipp Erhart, Dittmar Böckler, Patrick Schuler, Peter K. Plinkert, Ralph Hohenberger

**Affiliations:** ^1^ Department of Otorhinolaryngology, Head and Neck Surgery Heidelberg University Hospital Heidelberg Germany; ^2^ Department of Otorhinolaryngology, Head and Neck Surgery Augsburg University Hospital Augsburg Germany; ^3^ Department of Vascular and Endovascular Surgery Heidelberg University Hospital Heidelberg Germany

**Keywords:** head and neck paraganglioma, paraganglioma, quality of life

## Abstract

**Background:**

Head and neck paragangliomas (HNPGLs) are rare neuroendocrine tumors and often arise in the head and neck. Due to their localization, the tumor and its surgical treatment pose a risk for cranial nerve impairments. Few studies have focused on the health‐related quality of life (HRQOL) in patients with HNPGLs and their relation to tumor localization.

**Methods:**

In patients (*n* = 125) treated with primary surgery for HNPGLs between 2006 and 2023, clinical data was obtained. Long‐term QOL was assessed with the validated German version of the EORTC QLQ‐H&N43 with a mean follow‐up since surgery of 6.0 years.

**Results:**

Most common were carotid body PGLs (*n* = 78; 62.4%) including Shamblin I (*n* = 25), II (*n* = 42) and III (*n* = 9) along jugular (*n* = 31; 24.8%) and vagal (*n* = 10; 8.0%) tumors. In the QLQ‐H&N43, the scales fear of progression (41.2), coughing (33.9), neurological problems (22.4), sexuality (21.4), and swallowing (21.2) showed the highest mean scores. Jugular and vagal tumors showed higher symptom levels compared to carotid body tumors, especially Shamblin I.

**Conclusions:**

Surgical treatment of PGLs may lead to significant impairments in physical and psychological domains, especially in larger carotid body, vagal and jugular tumors. Structured pre‐ and postoperative cranial nerve examinations and interprofessional support should be provided to mitigate postoperative QOL reduction.

**Abbreviations**
CT: computed tomography
EORTC: European Organization for Research and Treatment of Cancer
HNPGL: Head and neck paraganglioma
MRI: magnetic resonance imaging
QLQ: quality of life questionnaire
QOL: quality of life
SDH: succinate dehydrogenase
WHO: World Health Organization

## 1 Introduction

Head and neck paragangliomas (HNPGLs) are rare neuroendocrine tumors that arise in the parasympathetic or sympathetic ganglia and represent 65%–70% of all PGLs [1]. They can be categorized into the most common sporadic type, the hyperplastic type in the context of chronic hypoxia and the hereditary type, which is characterized by earlier onset and more frequent bilateral and multilocular growth [2]. Genetic variations of the succinate dehydrogenase (SDH) gene contribute to this entity [3]. The WHO Classification of Tumors (4th edition) no longer differentiates between histologically benign or malignant PGLs, as any lesion has the potential for metastatic spread, which is reported in 6%–13% of cases [4, 5, 6].

HNPGLs (also referred to as “glomus tumors”) can further be classified by their site of manifestation in carotid, vagal, jugular, or tympanic [6, 7, 8]. In all locations, surgery has been the mainstay of primary therapy to histologically confirm the diagnosis and for complete tumor resection. However, surgery often poses a challenge due to the tumors' proximity to large blood vessels and cranial nerves. In carotid body PGLs, the focus is on vascular injury and reconstruction, whereas in vagal or jugular PGLs, neural dysfunction is the main challenge. Besides preoperative impairment of nerval function by the tumor itself, surgery may lead to further deficits of the cranial nerves and significant morbidity [9]. The decision between further wait and scan, tumor resection and definitive or adjuvant radiotherapy is challenging due to the slow tumor growth but higher risk for increased postoperative morbidity with increasing tumor volume [10]. Despite these pre‐ and postoperative impairments in patients with HNPGLs, their impact on health‐related quality of life (HRQOL) is little investigated. This study aimed to evaluate the HRQOL in a large patient cohort of surgically treated HNPGLs using the updated European Organization for Research and Treatment of Cancer (EORTC) Quality of Life Questionnaire with its updated specific head and neck module (QLQ H&N43) [11]. This manuscript further presents an interdisciplinary consensus of the authors on pre‐ and postoperative clinical examinations and recommendations for the management of tumor‐related nerve lesions to preserve the patients' QOL.

## 2 Patients and Methods

### 2.1 Ethical Considerations

The Ethics Committee of the Medical Faculty at the University of Heidelberg granted permission to conduct the study (Project S‐812/2022) according to the Declaration of Helsinki on biomedical research involving human subjects. The QOL questionnaires were retrospectively completed voluntarily by all participants after surgical treatment. All data were pseudonymized before analysis.

### 2.2 Patient Recruitment and Clinical Data

A retrospective chart review from 2006 to 2023 was conducted on patients with histologically confirmed HNPGLs at the Department of Otorhinolaryngology, Head and Neck Surgery and the Department of Vascular and Endovascular Surgery at the Heidelberg University Hospital. Exclusion criteria were patients under 18 years of age, other definitive histology than paraganglioma or primary radiation. Patients with isolated tympanic tumor localization were excluded, due to the different risk profile and surgical approach. In total, 125 patients met the inclusion criteria and underwent surgical therapy due to typical local symptoms, tumor growth, or nerval impairments. Patients without surgical intervention were excluded from this study. Nerval impairments prior and after surgery were routinely evaluated by clinical assessments in the Department of Otorhinolaryngology, Head, and Neck Surgery. These included the evaluation of the facial nerve (VII), glossopharyngeal nerve (IX), vagus nerve (X), accessory nerve (XI) and the hypoglossal nerve (XII) by inspection of the oropharynx, evaluation of muscular movement of the face, tongue, shoulder and the vocal cords as indicator of vagus nerve palsy.

### 2.3 Quality of Life Measurements

Health‐related QOL was assessed in March 2024 using the validated German version of the EORTC QLQ H&N43. It is designed to assess typical complications of head and neck cancer and its therapy and was recently updated, now including 43 items [11].

The QLQ H&N43 incorporates 12 multi‐item scales to assess Pain in the mouth (PA), swallowing (SW), problems with teeth (TE), dry mouth and sticky saliva (DR), problems with senses (SE), speech (SP), body image (BI), social eating (SO), sexuality (SX), problems with shoulder (SH), skin problems (SK) and fear of progression (ANX).

In addition, there are seven single items: problems opening the mouth (OM), coughing (CO), social contact (SC), swelling in the neck (SN), weight loss (WL), problems with wound healing (WO), and neurological problems (NE). As described in the EORTC scoring manual, all scales of the QLQ‐H&N43 were linearly transformed, to a range from zero to 100. Higher numbers indicate higher symptoms and lower QOL. The calculation of scores was implemented according to the scoring manual.

### 2.4 Statistics

All statistical analyses were performed with the Statistical Package for Social Sciences software, version 29.0.2 (SPSS, Chicago, IL).

Mean values were specified with standard deviation (SD). Mann–Whitney U test and Student's t‐test were used for the comparison between groups according to the nature of variables. Fisher's exact test, chi‐square test, and Kruskal–Wallis test were used for correlation analyses according to the nature of variables. The QLQ‐H&N43 scores were compared with a chi‐square test. Associations between the QOL scores and study variables were assessed by a Student's t‐test or variance analysis (ANOVA), after performing Levene's test for equal variances. A two‐sided *p* value of < 0.05 was considered statistically significant.

## 3 Results

### 3.1 Patients, Treatment and Clinical Characteristics

A total of 125 patients were included in the study. Characteristics and tumor localizations are depicted in Table 1. The most common subtype was carotid body paraganglioma (*n* = 78; 62.4%) with mostly Shamblin type II (*n* = 42; 53.8%). Twelve patients (9.6%) had bilateral PGLs. In the whole cohort, the mean maximal diameter based on preoperative imaging was 3.4 cm (range 0.8–10.0), and the mean tumor volume was 28.1 cc (range, 1.6–119.7). Jugular tumors (35.1 cc) had the largest mean tumor volume, followed by vagal (27.3) and carotid tumors (26.5). In seven patients, elevated uptake suggestive of distant metastasis was detected and interpreted as malignancy.

**TABLE 1:** Clinical characteristics and tumor localizations of the study cohort.

		Total	Carotid	Jugular	Vagal	Multiple	Other*
Patients		125	78 (62.4%)	31 (24.8%)	10 (8.0%)	5 (4.0%)	1 (0.8%)
Age (mean, range)			52.4 (19–84)	59.3 (32–78)	57 (29–75)	38.4 (28–65)	43
Sex	Male	39 (31.2%)	24 (30.8%)	10 (32.3%)	2 (20.0%)	3 (60.0%)	0
	Female	86 (68.8%)	54 (69.2%)	21 (67.7%)	8 (80.0%)	2 (40.0%)	1 (100%)
Tumor size (max. diameter)	cm	3.4	3.4	3.4	3.7	3.8***	x
Tumor volume (mean)	cc	28.1	26.5	35.1	27.3	11.7***	x
Shamblin Type		I	25 (32.1%)				
		II	42 (53.8%)				
		III	9 (11.5%)				
		ns**	2 (2.6%)				
Malignancy		7	5 (4.0%)	1 (0.8%)			1 (0.8%)

* Cervical vertebrae.

** Not specified**.

*** Largest tumor in multiple PGLs.

All patients received primary transcervical surgical therapy with the aim of complete tumor resection by an experienced vascular or ENT surgeon or in an interdisciplinary team. Preoperative angiography and an attempt at occlusion of the common carotid artery were conducted in 42.4% of cases; tumor embolization was performed in 46 patients (36.8%). Three (2.5%) of the patients received adjuvant radiation therapy. Mortality in the study cohort was 0%.

### 3.2 Preoperative Nerve Deficits

In total, 15 patients (12.0%) presented with preoperative cranial nerve deficits (Table 2). The most common was vagal nerve palsy (*n* = 11, 8.8%). Seven patients presented with multiple nerval deficits. Additionally, one patient had abducens nerve palsy. Preoperative cranial nerve palsy occurred in 20% of tumors with multiple localizations. The difference between nerval deficits in terms of tumor localization was statistically significant (*p* = 0.005).

**TABLE 2:** Pre‐ and postoperative cranial nerve deficits.

	Pre‐operative		Short‐term*		Long‐term**		Carotid		S I	S II		S III		Jugular		Vagal		Multil‐locular	
		%		%		%		%			%		%		%		%		%
VII	1	0.8	6	4.8	6	4.8	0	0	0	0	0	0	0	6	4.8	0	0	0	0
IX	5	4.0	6	4.8	9	7.2	5	4.0	0	4	3.2	1	0.8	2	1.6	1	0.8	1	0.8
X	11	8.8	18	14.4	27	21.6	10	8.0	0	8	6.4	2	1.6	8	6.4	7	5.6	2	1.6
XI	3	2.4	2	1.6	2	1.6	1	0.8	0	1	0.8	0	0	1	0.8	0	0	0	0
XII	6	4.8	16	12.8	9	7.2	4	3.2	0	2	1.6	2	1.6	2	1.6	3	2.4	0	0
Sympathetic ganglion	1	0.8	2	1.6	2	1.6	2	1.6	0	2	1.6	0	0	0	0	0	0	0	0
Total patients	15	12.0	24	19.2	34	27.2	11	14.1	0	9		2		14	45.2	7	70	2	40

* Defined as nerve damage documented at any time postoperatively, but recovered within 6 months.

** Defined as persistent nerve damage after 6 months, S = Shamblin‐classification.

### 3.3 Complications and Morbidity

Thirteen patients (10.4%) had severe complications, which were either surgical, due to angiography with embolization, or general in nature. Complications occurred mostly in patients with multilocular tumor localization (40%). These included one patient with bilateral vagal injury, who experienced extreme blood pressure fluctuations postoperatively. Another required placement of a percutaneous gastrostomy tube due to severe swallowing difficulties. In one such case, temporary tracheostomy due to laryngeal aspiration was necessary, and one had a stroke. Following angiography and embolization, one patient experienced a cholesterol embolism. Other complications not related to surgery or intervention included one cardiac arrest and one catheter‐associated dissection of the abdominal aorta. There were no significant differences in severe complications regarding tumor localization (*p* = 0.24) or mean tumor size (*p* = 0.432).

In total, 58 patients (46.4%) presented with an impairment of at least one cranial nerve at any time after surgery. Twenty‐four (19.2%) patients showed temporary paralysis of cranial nerves that resolved during the follow‐ups in the first 6 months. A total of 34 patients (27.2%) exhibited cranial nerve palsies that persisted for a minimum of 6 months following the surgery (Table 2). Most common was an impairment of the vagal nerve (21.6%).

#### 3.3.1 Carotid Body PGLs

Preoperative cranial nerve palsy occurred in 5.1% of carotid PGLs. Surgical complications occurred in 10.2% of patients with carotid body tumors. Five patients experienced postoperative bleeding from the carotid artery. One patient suffered a stroke intraoperatively. Another patient needed a temporary tracheostomy due to laryngeal aspiration. Postoperative permanent cranial nerve deficits occurred in 14.1% of carotid body PGLs (Shamblin I: 0%; II: 21.4%; III: 22.2%).

#### 3.3.2 Jugular PGLs

Preoperative cranial nerve palsies were most frequently observed in jugular tumors (22.6%). Postoperative complications occurred in 9.7% and included one patient with a cerebrospinal fluid leak due to skull base injury, which was subsequently occluded. Two patients required the placement of a percutaneous gastrostomy tube due to severe swallowing difficulties. Permanent postoperative cranial nerve deficits occurred in 45.2% of cases in jugular PGLs.

#### 3.3.3 Vagal PGLs

Preoperative cranial nerve palsy occurred most frequently in vagal (20.0%) PGLs; no major surgical complications occurred. Vagal PGLs accounted for the majority of permanent postoperative cranial nerve deficits (70.0%).

### 3.4 Quality of Life Assessments

The response rate for the QOL assessments was 51.2% (64 of 125) with a mean follow‐up since surgery of 6.0 years (SD ± 4.5; range 0.3–16.2). Multi‐item and single‐item scales according to tumor localization and nerve palsies are depicted in Tables 3 and 4.

**TABLE 3:** Multi‐item scales of the EORTC QLQ‐H&N43.

Factors	*N*	PA	SW	TE	DR	SE	SP	BI	SO	SX	SH	SK	ANX
All (±SD)	64	12.1 (16.3)	21.2 (26.2)	14.1 (16.2)	22.7 (22.9)	16.1 (24.7)	19.1 (26.6)	16.8 (23.7)	19.1 (26.6)	21.4 (31.9)	20.3 (27.9)	10.9 (16.0)	41.4 (33.2)
Site*													
Carotid body	40	13.8 (18.8)	15.4 (22.1)	12.2 (14.4)	23.3 (25.5)	13.8 (23.5)	24.7 (27.8)	17.8 (25.0)	13.0 (20.0)	20.8 (31.7)	15.4 (23.1)	10.6 (15.5)	40.0 (33.5)
Jugular	17	8.3 (11.4)	27.9 (32.4)	19.0 (19.9)	21.5 (17.4)	21.6 (29.3)	36.5 (34.7)	17.6 (24.6)	28.9 (33.6)	21.6 (32.7)	31.4 (34.8)	11.1 (18.0)	44.1 (35.8)
Vagal	6	11.1 (8.6)	40.3 (25.5)	11.1 (17.2)	22.2 (22.8)	11.1 (13.6)	46.7 (28.3)	7.4 (11.5)	29.2 (37.2)	16.7 (33.3)	13.9 (26.7)	13.0 (17.8)	44.4 (31.0)
*p*		0.523	0.044	0.334	0.965	0.494	0.148	0.608	0.071	0.948	0.116	0.944	0.894
Pre‐op nerve palsy													
No	55	13.2 (16.9)	16.8 (22.7)	12.5 (15.1)	23.3 (23.9)	15.2 (25.1)	25.8 (27.8)	17.8 (25.0)	14.6 (21.8)	19.1 (30.3)	20.6 (27.4)	11.9 (16.8)	41.2 (33.3)
Yes	9	5.6 (9.3)	48.1 (31.7)	23.5 (20.4)	18.5 (15.5)	22.2 (22.0)	54.1 (33.6)	11.1 (12.4)	46.3 (37.3)	35.2 (39.5)	18.5 (32.7)	4.9 (8.1)	42.6 (34.5)
*p*		0.194	0.001	0.030	0.441	0.430	0.008	0.438	0.035	0.162	0.837	0.228	0.909
Post‐op nerve palsy													
No	35	10 (11.0)	18.3 (26.3)	14.0 (17.7)	16.2 (17.4)	11.9 (22.7)	22.9 (28.7)	12.7 (17.7)	19.4 (29.0)	10.0 (30.5)	15.7 (27.4)	16.2 (26.0)	35.2 (31.8)
Yes	29	14.7 (20.9)	24.7 (26.1)	14.2 (14.5)	30.5 (26.4)	21.3 (26.3)	38.1 (30.1)	21.8 (28.9)	18.7 (24.0)	23.0 (34.0)	25.9 (28.0)	14.9 (26.1)	48.9 (33.9)
*p*		0.285	0.337	0.960	0.016	0.132	0.042	0.144	0.919	0.712	0.149	0.890	0.103

Abbreviations: ANX, fear of progression; BI, body image; DR, dry mouth and sticky saliva; PA, pain in the mouth; SE, problems with senses; SH, problems with shoulder; SK, skin problems; SO, social eating; SP, speech; SW, swallowing; SX, sexuality; TE, problems with teeth.

* One patient had multilocular growth/growth in the vertebral spinae.

**TABLE 4:** Single‐item scales of the EORTC QLQ‐H&N43.

Factors	No	OM	CO	SC	SN	WL	WO	NE
All patients	64	17.2 (30.3)	33.9 (34.4)	17.7 (30.3)	15.6 (25.9)	7.8 (22.0)	6.8 (18.0)	22.4 (32.0)
Site	40	15.8 (28.2)	28.3 (31.6)	18.3 (31.1)	16.7 (27.2)	6.7 (20.3)	5.0 (17.8)	21.7 (33.4)
Carotid body								
Jugular	17	23.5 (38.7)	43.1 (36.8)	21.6 (33.2)	13.7 (23.7)	5.9 (24.3)	11.8 (20.2)	25.5 (32.3)
Vagal	6	11.1 (17.2)	44.4 (45.5)	5.6 (13.6)	11.1 (27.2)	22.2 (27.2)	5.6 (13.6)	11.1 (17.2)
*p*		0.599	0.250	0.545	0.852	0.254	0.435	0.642
Pre‐op nerve palsy								
No	55	19.4 (31.9)	32.1 (34.5)	15.8 (30.0)	13.9 (24.6)	6.7 (21.7)	7.3 (18.9)	23.6 (33.1)
yes	9	3.7 (11.1)	44.4 (33.3)	29.6 (30.9)	25.9 (32.4)	14.8 (24.2)	3.7 (11.1)	14.8 (24.2)
*p*		0.009	0.323	0.205	0.200	0.307	0.585	0.448
Post‐op nerve palsy								
No	35	17.1 (31.7)	32.4 (33.8)	13.3 (28.2)	16.2 (26.0)	8.6 (21.9)	6.7 (19.5)	21.0 (33.3)
Yes	29	17.2 (29.0)	35.6 (35.6)	23.0 (32.2)	14.9 (26.1)	6.9 (22.5)	6.9 (16.4)	23.0 (31.0)
*p*		0.990	0.710	0.206	0.849	0.103	0.960	0.894

Abbreviations: CO, coughing; NE, neurological problems; OM, problems opening the mouth; SC, social contact; SN, swelling in the neck; WL, weight loss; WO, problems with wound healing.

In the whole cohort, the highest symptom burden was reported in the multi‐item scales Fear of progression (41.4 ± 33.2), Dry mouth (22.7 ± 22.0), Sexuality (21.4 ± 31.9) and Swallowing (21.2 ± 26.2). The single‐item scales with the highest symptom burden were Coughing (33.9 ± 34.4) and Neurological problems (22.4 ± 32.0).

In carotid body tumors, coughing (28.3), speech (24.7), dry mouth (23.3), and neurological problems (21.7) were the highest functional implications. In jugular tumors, speech (36.5), problems with shoulder (31.4), swallowing (27.9), and social eating (28.9) were the highest scales. In vagal tumors, respectively coughing (44.4), swallowing (40.3), speech (46.7), and social eating (29.2).

In the domains swallowing, speech, social eating, and coughing, differences between the localization subgroups were evident: While patients with carotid body tumors showed the lowest impairments, jugular and vagal tumor patients showed higher functional impairments with a statistically significant difference in Swallowing (15.4 vs. 27.9 vs. 40.3, *p* = 0.04).

The presence of preoperative nerve palsies had a significant impact on the reported HRQOL. The domains swallowing (16.8 vs. 48.1; *p* = 0.001), dental problems (12.5 vs. 23.5; *p* = 0.03), speech (25.8 vs. 54.1; *p* = 0.008), and social eating (14.6 vs. 46.3; *p* = 0.035) were significantly higher if preoperative nerve palsies were present. The patients with postoperative nerve deficits reported worse HRQOL in all multi‐item scales compared to patients without nerve deficits, with statistically significant differences in the categories dry mouth (16.2 vs. 30.5; *p* = 0.016) and speech (22.9 vs. 38.1; *p* = 0.042).

Twenty‐six (20.8%) patients experienced local recurrence within a median follow‐up interval of 27 months. Of these, nine (34.7%) were carotid tumors, twelve (46.2%) were jugular, two were vagal (7.7%), two were multilocular (7.7%), and one was atypically located in the cervical vertebrae.

In the QOL comparison of patients with recurrence vs. no recurrence, only the category weight loss showed a statistical difference (19.0 vs. 4.7, *p* = 0.03). In patients with QOL data and with metastatic disease (*n* = 5), no relevant differences in mean scores were evident. In patients with bilateral PGL (*n* = 5) vs. single location only the categories opening mouth (18.6 vs. 0), weight loss (26.7 vs. 6.2) and problems with senses (30.0 vs. 15.0) showed relevant differences in the mean score; due to the small groups, further statistical analysis was considered not adequate.

## 4 Discussion

### 4.1 Key Findings

**FIGURE 1:**
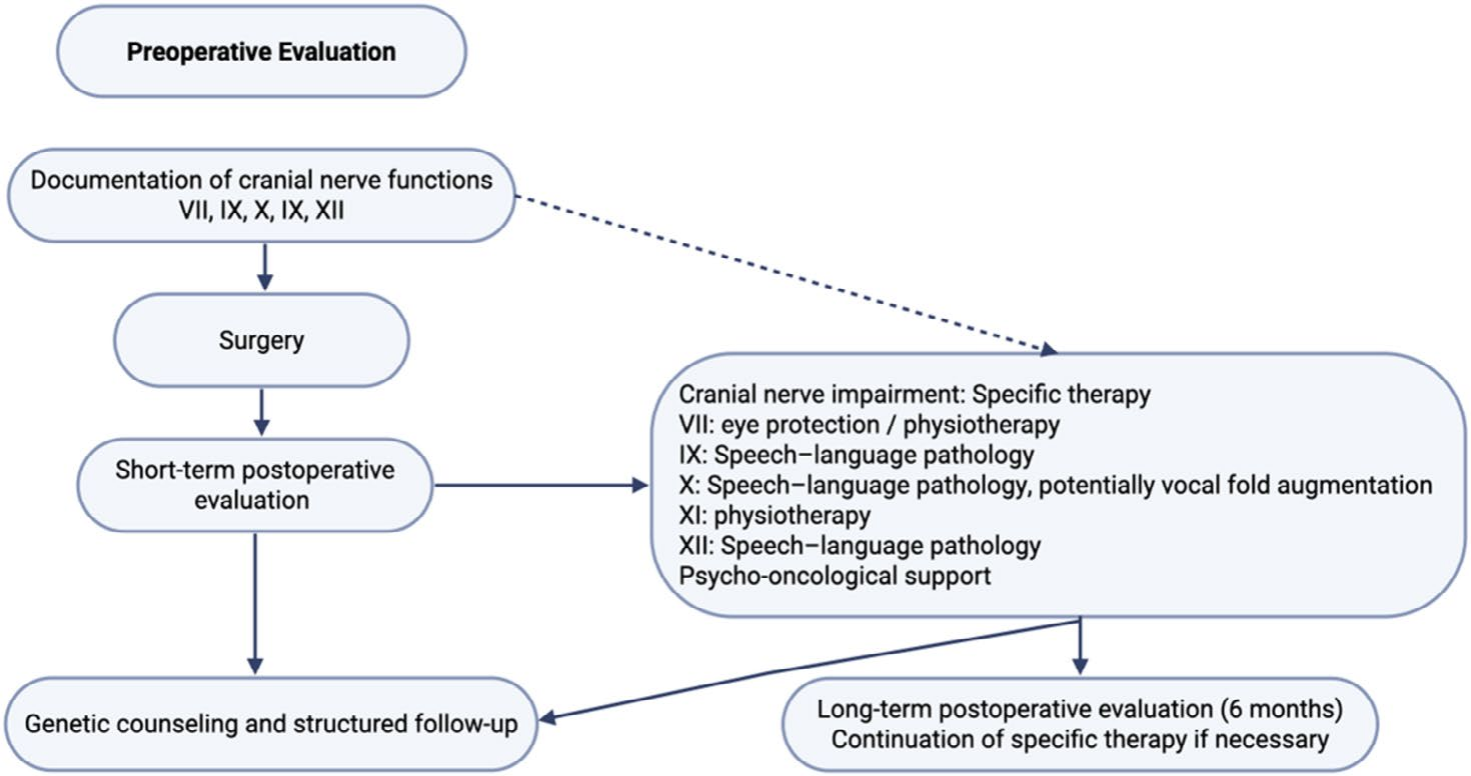
Algorithm to routinely evaluate cranial nerve function and initiate specific therapy to mitigate quality of life impairments. [Color figure can be viewed at wileyonlinelibrary.com]

This study evaluated the long‐term HRQOL in 125 patients with surgically treated HNPGLs using the EORTC QLQ H&N43 questionnaire. It revealed clinically relevant impairments in multiple domains of daily life; like fear of tumor progression, persistent coughing and multiple oral functions, especially when cranial nerve damages are present. Patients with jugular and vagal tumors showed higher symptom scores compared to carotid body tumors; removal of smaller carotid body tumors is possible without QOL impairments. This is one of the largest cohorts of HNPGLs with long‐term follow up and the first structural assessment of their HRQOL. In Figure 1 the authors present an interdisciplinary recommendation to routinely assess cranial nerve function prior to and after HNPGL treatment to specifically and early address functional impairments and sustain a high QOL in HNPGL patients.

### 4.2 Strengths and Limitations

Due to the retrospective nature of our study, we have not collected longitudinal data on patient characteristics, disease, and QOL. The questionnaires were collected at a single timepoint with a large discrepancy of postoperative follow‐up. Structured pre‐ and postoperative assessments would be an ideal design for a more comprehensive understanding of the QOL implications of HNPGLs. There is no dedicated questionnaire for QOL measurement in HNPGLs. Given the broad similarity of symptoms and considering the psychological implications of both diseases, we have considered the QLQ H&N43 to be the best fit, but some specific symptoms may not have been adequately addressed. The development and validation of a specific patient‐reported outcome measure may be a reasonable aim for the future. Further, for the QLQ H&N43, there is no healthy reference population to compare the symptom levels. As a strength of this study, we consider the large patient cohort. To the best of our knowledge, it is also the first study to utilize the recently revised version of the QLQ H&N43 in this patient population.

### 4.3 Treatment and Diagnostic Algorithm for HNPGLs

Surgical treatment is the mainstay for curative therapy of HNPGLs. All patients included in this study underwent surgical therapy with the objective of complete tumor resection. In this study, preoperative tumor embolization and/or temporary occlusion of the carotid artery was performed in selected patients. In which cases these procedures are beneficial to minimize blood loss and reduce the incidence of surgical complications is under discussion [2]. Ellis et al. demonstrated that patients with carotid body PGL and SDHB sequence variation should undergo preoperative embolization before resection, given the higher malignant potential associated with this condition [12]. In current reviews, success rates of 80% or more were reported in achieving tumor devascularization, with complication rates below 4% and hence facilitating surgical resection in well‐selected patients with Shamblin III or vagal/jugular tumors [13, 14]. The authors consent with the recommendation to evaluate preoperative occlusion of the carotid artery and tumor embolization not routinely, but in selected large tumors.

In asymptomatic patients with no signs of malignancy, observation with regular imaging and clinical assessment of cranial nerve function is also a viable option. Additionally, other authors have reported a low toxicity and good local control with primary radiotherapy in the management of head and neck paragangliomas [8, 15, 16].

In a study conducted by Künzel et al., the functional results and long‐term tumor control rate were compared in patients who had undergone treatment by surgery, radiotherapy and observation. In the surgical group, 20% of patients experienced permanent nerve paralysis, while the tumor control rate was 100% in both the surgical and radiotherapy groups. Only one patient who had been observed clinically demonstrated tumor progression [17].

The primary determinants of inherited HNPGLs are a documented familial history of these tumors, a history of adrenal or extra‐adrenal paragangliomas, multifocality or additional clinical presentations that are also associated with SDH sequence variants [18]. SDHD‐associated paragangliomas are frequently benign and multilocular, whereas tumors with SDHB alterations, tumor size of > 5 cm and extra‐adrenal location are more prone to metastasize [19, 20, 21, 22]. Therefore, SDH enzyme complex mutation analysis and genetic counseling should be offered to all patients [23, 24].

### 4.4 Quality of Life in HNPGLs

The study cohort showed impaired QOL in many scales of the EORTC QLQ‐HN43. The scale Fear of progression showed the highest mean score with 41.4 (±33.2), highlighting the psychological impact of this disease. Further, the scales coughing (33.9 ± 34.4), neurological problems (22.4 ± 32.0), dry mouth (22.7 ± 22.0), and sexuality (21.4 ± 31.9) demonstrated the highest functional impairments. In jugular PGLs, also Problems with shoulder (31.4 ± 34.8) and in jugular and vagal tumors, swallowing, speech, and social eating were the scales with the highest mean scores, while surgery of carotid body tumors was less impairing for the patients. Analyses also demonstrated that QOL was worse when pre‐ or postoperative nerve palsies were present (e.g., Swallowing: 16.8 ± 22.7 vs. 48.1 ± 31.7, *p* < 0.001).

The mean scores in this cohort are comparable to patients with common head and neck carcinomas including oral, laryngeal or hypopharyngeal tumors acquired 3 months post‐treatment. In particular, the domains Fear of progression, shoulder problems, swallowing, coughing, and neurological problems are comparable in both cohorts [11]. The clinically relevant impairment in the HNGPL patients is demonstrated by the fact that the minimal clinically important difference for the exemplary domain swallowing was calculated between 10 and 14 [25].

Only few studies have so far addressed the health‐related QOL in patients with HNPGLs. Obholzer et al. used seven different questionnaires and demonstrated globally impaired QOL in their study of 60 patients with head and neck paraganglioma treated with different treatment options [26]. Havekes et al. used the Hospital Anxiety and Depression Scale, Multidimensional Fatigue Index (MFI‐20), Short Form‐36 (SF‐36), and Nottingham Health Profile (NHP) in 82 patients with HNPGLs without specification of treatment [9]. They also showed that higher fatigue and impairment in physical functioning were present, and dysphonia was highlighted in functional implications.

The EORTC QLQ‐C30 and H&N 35 was used to demonstrate altered QOL in 20 patients who underwent radiotherapy by Galland‐Girodet et al. [27]. Further cohorts of 26 and 53 patients who underwent radiotherapy for jugular PGL were investigated with the generic questionnaires SF‐12 and SF‐36 and showed excellent tumor control and little QOL impairment due to the radiotherapy [28, 29]. In summary, neither the EORTC H&N 35 nor its updated version H&N43 have been used in surgically treated HNPGL patients and prior patient cohorts are smaller compared to this study.

As HNPGLs are a rare disease, there is a lack of clinical guidelines. Data on these tumors is still sparse and relies on single‐center experiences. A prospective registry with prospective collection of clinical and QOL data is an essential beginning to further improve the management of these tumors as recently initiated by de Bresser et al. [30].

## 5 Conclusion

Surgical treatment of HNPGLs can lead to significant, long‐term impairments in HRQOL not only in physical (mainly oropharyngeal functions), but also psychological domains (fear of tumor progression). The most relevant factor on QOL is the tumor localization, as especially jugular and vagal tumors show a higher incidence while carotid body tumors may be resectable with little to no postoperative impairments. QOL should be given a high priority in the interdisciplinary treatment decision. Genetic counseling, pre‐ and postoperative cranial nerve assessments and specific therapy in case of functional impairments should be implemented in all patients.

## Author Contributions

All authors made substantial contributions to the study. C.S. participated in data acquisition and analysis, interpreted the data, drafted the manuscript. P.E. designed and coordinated the study, participated in data acquisition, critically revised the manuscript for important intellectual content. P.S. revised the manuscript, interpreted data. D.B. revised the manuscript, interpreted data, critically revised the manuscript for important intellectual content. R.H. designed and coordinated the study, participated in data acquisition and analysis, drafted the manuscript, interpreted data.

## Ethics Statement

The Ethics Committee of the Medical Faculty at the University of Heidelberg granted permission to conduct the study (Project No. S‐812/2022).

## Consent

The questionnaires were completed voluntarily by all participants.

## Conflicts of Interest

The authors declare no conflicts of interest.

## Data Availability

The data that support the findings of this study are available from the corresponding author upon reasonable request.
